# Healthy Lifestyles of University Students in China and Influential Factors

**DOI:** 10.1155/2013/412950

**Published:** 2013-07-09

**Authors:** Dong Wang, Xiao-Hui Xing, Xian-Bo Wu

**Affiliations:** ^1^School of Public Health and Tropical Medicine, Southern Medical University, Guangzhou 510515, China; ^2^Department of Development & Planning, Southern Medical University, Guangzhou 510515, China

## Abstract

This study was conducted to analyze to what extent university students exhibit healthy lifestyles and which sociodemographic variables influence healthy lifestyles. 4809 university students randomly selected were measured by use of the Healthy Lifestyle Scale for University Students questionnaire. When controlling for the other variables, the total healthy lifestyles score was predicted by gender, grade, father's level of education, and type of institution; exercise behaviour was partially predicted by gender, grade, type of institution, and family monthly income; regular behaviour was modulated by gender, grade, type of institution, family monthly income, and father's educational level; nutrition behaviour was partially affected by type of institution, family monthly income, and father's educational level; health risk behaviour was modulated by gender, mother's level of education, and family monthly income; health responsibility was modulated by gender, grade, type of institution, and father's educational level; social support was modulated by gender, grade, and father's educational level; stress management was modulated by gender, grade, type of institution, and mother's education level; life appreciation was modulated by grade, type of institution, and mother's educational level. These influences should be taken into account in designing interventions for specific socio-demographic profiles that might be at higher risk for certain behaviours.

## 1. Introduction

In recent decades, lifestyle has been recognised as an important determinant of health status and has become a focus of increasing research interest worldwide. The World Health Organization (WHO) has stated that 60% of an individual's health-related quality of life depends on his/her lifestyle [1]. Numerous publications [2–4] have shown that healthy lifestyle practices reduce disease occurrence and mortality rates and socio-demographic dimensions such as sex, age, marital status, economical level, and paid employment correlate with healthy lifestyle [5].

Healthy lifestyles depend on the early adoption of healthy living habits; unhealthy lifestyles among youths are strongly linked to unhealthy habits in adulthood [6, 7]. Health-related behaviours in early stages of life affect the disease risks related to lifestyle in later periods of life. Although it is difficult to change unhealthy habits that adults have adopted in their youth, many effects of health risk factors among adults are avoidable if these behaviours are identified and changed at an early stage [8]. Therefore, it is important to increase healthy lifestyle behaviours among young people.

University students represent a major segment of the young adult population [9]. They typically enter a dynamic transitional period of new independence from their parents that is characterised by rapid, interrelated changes in body, mind, and social relationships [10] and experience a new environment that generally involves increased workload and stress, altered patterns of life, which are significant contributors to unhealthy lifestyles. Previous studies [11–13] on healthy lifestyles indicate that majority of university students are minimally engaged in health-promoting behaviours and exhibit behavioural health risks, such as tobacco use, alcohol and substance abuse, and improper diet and physical activities [14, 15]. Some diseases such as sexually transmitted diseases [16], prehypertension [17], psychological symptoms and mental illness [18], and obesity and being overweight [19] are also on the rise among college students. These behaviour patterns and their consequences typically persist into adulthood, jeopardising individuals' health status in later life [20]. Many studies have shown that the health behavior is modulated by the socio-demographical variables, such as gender, age, socio-economic level, ethics, examination level at admission, and educational level of their parents [14, 21–24].

Health education in the university environment is an ideal and cost-effective means of developing healthy lifestyles, because university students are in a unique stage of knowledge absorption and personality shaping. In the educational study carried out by Hsiao et al. [25], significant increases were reported in the total and subscale score averages of healthy life style behaviours. In addition, Yeh et al. [26] reported positive changes in the healthy lifestyle behaviours of students after the education given to students to increase their healthy lifestyle behaviours. University students both constitute a large part of the young population and are the primary target population for education about the importance of healthy lifestyles; it is essential for the promotion of their healthy growth to investigate their healthy lifestyle behaviour and influential factors. 

The development of higher education in China has been very dramatic in recent years. For example, there were 5.56 million university students in 2000; this number had tripled (to 15.62 million) in 2005 and is expected to increase to 33.5 million by 2015 [27]. Extensive evidence has indicated that university students in China engage in health-risk behaviours such as smoking, drinking alcohol, lack of exercise, lack of sleep, and poor eating habits [28–31].

All these results on the healthy lifestyles and influential factors of university students are primarily from North American, Mexican, Hong Kong, and Taiwan universities. In mainland China, research on the lifestyles of university students has focused primarily on the relationship between health status and health risk behaviours, few studies have quantified healthy lifestyles among these students, and there are particularly less information to date that has been made into the influential factors of the healthy lifestyles in this population.

To study what promotes healthy behavior among university students, the current study was conducted to determine the level of university students' application of healthy lifestyle behaviours and affecting socio-demographic factors by use of the Healthy Lifestyle Scale for University Students (HLSUS) [32] for evaluating the healthy lifestyles for university students.

## 2. Materials and Methods

### 2.1. Sample and Selection

This descriptive and cross-sectional study used a two-stage, stratified sampling method. First, we applied a proportionate allocation strategy to sample 9 universities, which were selected randomly according to geographic location to provide a representative nationwide sample. Next, 600 students in each selected university were selected randomly by determining the number of students that could be obtained from the student lists of these schools. A total of 5,400 university students of 9 universities located throughout mainland China were recruited for participation, 5,126 out of the total of 5400 selected subjects agreed to be recruited for this investigation. The overall response rate was 94.93%.

Prior to conducting this study, ethical approval was obtained from the Area Health Service Ethics Committee and the Human Ethics Committee of Southern Medical University. All students to be included in the research were informed about the objective of the research before the application of forms, written informed consent was received from the students, confidentiality principle was observed, and each respondent was free to discontinue participation at any time.

### 2.2. Questionnaire

The questionnaire included descriptive information and the Healthy Lifestyle Scale for University Students (HLSUS). Descriptive information was collected on the socio-demographic characteristics of students (age, gender, grade, education level of their parents, family monthly income level, residence before enrolled in school, location of institution, and type of institution).

The HLSUS was developed based on the Pender's Health Promotion Model, and a detailed description on the validity and reliability of the scale can be found in the previous publication [32]. It consisted of 38 items, which were divided into 8 dimensions: exercise behavior, regular behavior, nutrition behavior, health risk behavior, health responsibility, social support, stress management, and life appreciation. The frequency of reported behaviors was obtained using a self-reporting Likert scale with a five-point response format, “never, rarely, sometimes, usually, and always,” with the rating score ranging from 1 to 5. Each of the 38 items has a maximum possible score of 5 and a minimum possible score of 1. Items 13, 14, 15, and 16 are scored inversely so that a higher number indicates impairment (i.e., 1 = 5 − 4, 4 = 5 − 1, and 2 = 5 − 3, etc.). HLSUS is a self-administered instrument that takes approximately 8 minutes to complete. The total score obtained from the scale indicates the level of healthy lifestyle behaviours. The lowest total score is 38 and the highest is 190. A higher score indicates that the subject performs a higher level of the indicated behaviours.

### 2.3. Fieldwork

Because the study was intended to identify the usual pattern of university students' health practices and to avoid the confounding effects of seasonal holidays and the stressful examination period, the survey was conducted in the midsemester. To maximise the response rate and avoid researchers' influence on the respondents, all questionnaires were delivered and collected face-to-face by students whom the researchers had trained as interviewers. The respondents completed the questionnaires individually, and the interviewers were on site to explain any unclear items without inducement.

### 2.4. Data Management

All valid questionnaires were entered in duplicate into the database by two independent postgraduate students using EpiData software (version 3.1; EpiData Association, Odense, Denmark). Any discrepancy between the two operators was resolved by cross checking the duplicate data manually and by computer. Data from 218 subjects were excluded due to missing responses to more than five questions or to evidence that the respondent had not taken the questionnaire seriously (e.g., a score outside the normal variation or a majority of “always” or “never” responses). Thus, data from 4,908 valid questionnaires were analysed in this study. Missing values were replaced by mean item values.

### 2.5. Statistical Analysis

Statistical analysis was carried out by using SPSS for windows (Southern Medical University, China.release17). The statistical description of the socio-demographic variables was performed by using frequencies, percentages, means, and standard deviations. For univariate and multivariate analyses, some demographic characteristics were collapsed to increase numbers in each cell when frequencies were less than 5% of the sample. Parents' educational level was collapsed into three levels: primary, which included no education, secondary, and university. The type of institution was collapsed into three levels: three-year college, medical university, and comprehensive university. The grade was divided into three categories: freshmen, sophomores, and senior students (who had been in the university for 3~5 years). The location of institution was classified into three categories: East China, midland, and West China.

To analyse the relation between socio-demographic characteristics and healthy lifestyles, we computed *t*-tests for dichotomized variables and one-way ANOVAs for dimensions with more than two categories. To analyse whether the combined effect of the socio-demographic variables predicts health behaviour, we performed multiple regression analyses over each dimension of the HLSUS and over the total score. The variables that were significant in the univariate analyses were used as predictors. To compute the first model, all of the variables were entered, and nonsignificant variables were iteratively excluded from the model until it reached the point of best adjustment based on the *R* ^2^ value and significance of the variables included in the model. Since some variables had more than two categories, dummy variables were computed as follows. For father's and mother's educational level, we used reference primary education (primary = 1; secondary = 0; university = 0) and reference secondary education (primary = 0; secondary = 1; university = 0). Similarly, dummy variables were created for type of university: reference low level (three-year college = 1; medical university = 0; comprehensive university = 0) and reference medium level (three-year college = 0; medical university = 1; comprehensive university = 0).

## 3. Results

### 3.1. Subject Characteristics

The subjects in this study were 16–25 years (mean, 21.7 years) of age. Of the 4,908 students who completed the questionnaire, 2,636 (53.71%) were male and 2,272 (46.29%) were female. There were 2,356 freshmen, 1,285 sophomores, and 1,267 junior students and above. For more detail see Table 1.

### 3.2. Healthy Lifestyles of University Students

Table 2 shows the values of the HLSUS dimension scores. The HLSUS Scale score average of students was 139.84 ±17.07 (lower score = 38, upper score = 190).

### 3.3. Socio-Demographic Characteristics and Healthy Lifestyles

#### 3.3.1. Total Healthy Lifestyles Score

In the univariate analyses, total healthy lifestyles score was significantly related to grade, location of institution, type of institution, both parents' educational level, residence before being enrolled in school, and family monthly income. Healthier behaviors were observed in freshmen (*P* = 0.000), institution located in West China (*P* = 0.003), medical university (*P* = 0.000), and higher family monthly income (*P* = 0.01). Healthier behaviors were found in those students whose fathers and mothers had university degrees (*P* = 0.000; *P* = 0.000, resp.) and in those students who came from urban areas before being enrolled in school (*P* = 0.000). In the multiple regression analyses, gender, grade, father's level of education, and type of institution were the significant variables in the adjusted model, explaining 32.02% of the variance (*R* ^2^ = 0.3202; *P* < 0.005) (Table 4).

#### 3.3.2. Exercise Behavior

Male students were more active than female students (*P* = 0.000). Exercise behavior was also significantly related to grade, location of institution, type of institution, parents' educational level, residence before being enrolled in school, and family monthly income. In particular, freshmen students (*P* = 0.000) whose institution is located in West China (*P* = 0.000), whose university is medical university (*P* = 0.000), Whose parents had university education (*P* = 0.000; *P* = 0.000, resp.), those who had the highest family monthly income (*P* = 0.002), and those who came from urban areas before being enrolled in school (*P* = 0.000) reported more exercise behavior than the other groups. The variables included in the multivariate stepwise regression equation were gender, grade, type of institution, and family monthly income (*R* ^2^ = 0.4173; *P* < 0.05), and these variables explained 41.73% of the total variance for exercise behavior (Table 4).

#### 3.3.3. Regular Behavior

Female students were more regular than male students (*P* = 0.000). Grade, location of institution, type of institution, and father's educational level also were significantly related with regular behavior in the univariate analyses. Specifically, the highest scores were found among freshmen students (*P* = 0.002), whose institution is located in West China (*P* = 0.000), whose university is medical university (*P* = 0.000), and whose fathers (*P* = 0.029) had completed university education. The multiple regression model included type of institution, grade, family monthly income, gender, and father's educational level as predicting variables (*R* ^2^ = 0.2104; *P* < 0.05) and explained 21.04% of the total variance of the predicted variable (Table 4).

#### 3.3.4. Nutrition Behavior

In the univariate analysis with nutrition behavior as the dependent variable, differences were found for grade, location of institution, type of institution, parents' educational level, residence before being enrolled in school, and family monthly income. The highest scores were found among junior students and above (*P* = 0.000), those students whose institution located in East China (*P* = 0.000), those students whose university is medical university (*P* = 0.000), highest family monthly income (*P* = 0.000), those who came from urban areas before being enrolled in school (*P* = 0.000), and those whose parents (*P* = 0.000; *P* = 0.000, resp.) had completed university education. Multiple regression presented that type of institution, family monthly income, and father's educational level were included in the final equation and those accounted for 22.47% of the total variance (*R* ^2^ = 0.2247; *P* < 0.05) (Table 4).

#### 3.3.5. Health Risk Behavior

In the univariate analysis, the score of health risk behavior was significantly related to gender, grade, location of institution, both parents' educational level, residence before being enrolled in school, and family monthly income. Female students were less engaged in health risk behaviors than male students (*P* = 0.000). Little health risk behaviors were observed in sophomores (*P* = 0.031), institution located in midland China (*P* = 0.000), and low family monthly income (*P* = 0.000). Little health risk behaviors were also found in those students whose parents had primary educational level (*P* = 0.000; *P* = 0.000, resp.) and who came from rural areas before being enrolled in school (*P* = 0.000). In the multiple regression analyses, gender, mother's level of education, and family monthly income were the significant variables in the adjusted model, explaining 19.36% of the variance (*R* ^2^ = 0.1936; *P* < 0.005) (Table 4).

#### 3.3.6. Health Responsibility

In the univariate analysis with health responsibility behavior as the dependent variable, differences were found for gender, grade, type of institution, parents' educational level, and residence before being enrolled in school. The healthier health responsibility behaviors were observed in female students (*P* = 0.000). The highest scores were found among junior students and above (*P* = 0.001), those students whose university is medical university (*P* = 0.000), those who came from urban before enrolled in school (*P* = 0.000), and those whose father (*P* = 0.000) and mothers (*P* = 0.000) had completed university education. Multiple regression presented that gender, grade, type of institution, and father's educational level were included in the final equation, and those accounted for 9.22% of the total variance (*R* ^2^ = 0.0922; *P* < 0.05) (Table 4).

#### 3.3.7. Social Support

In the univariate analysis, the score of social support behavior was significantly related to gender, grade, type of institution, both parents' educational level, residence before being enrolled in school, and family monthly income. Female students had better social support behavior than male students (*P* = 0.000). Best support behaviors were observed in freshmen (*P* = 0.023), medical university (*P* = 0.000), higher family monthly income (*P* = 0.022), those students whose parents had completed university education (*P* = 0.000; *P* = 0.000, resp.), and those who came from urban areas before being enrolled in school (*P* = 0.000). In the multiple regression analyses, gender, father's level of education, and grade were the significant variables in the adjusted model, explaining 20.13% of the variance (*R* ^2^ = 0.2013; *P* < 0.005) (Table 4).

#### 3.3.8. Stress Management

In the univariate analyses, stress management was significantly related to all of the assessed socio-demographic characteristics. Better stress management strategies were found among male students (*P* = 0.000). Furthermore, freshmen (*P* = 0.001), those students whose institution located in East China (*P* = 0.000), those students whose university is medical university (*P* = 0.000), higher family monthly income (*P* = 0.000), those who came from urban before being enrolled in school (*P* = 0.000), and those whose fathers (*P* = 0.000) and mothers (*P* = 0.000) with higher educational levels presented better stress management strategies than the other groups. The multiple regression model included gender, grade type of institution, and mother's education in the final equation (*R* ^2^ = 0.2181; *P* < 0.05), and these variables explained 21.81% of the final variance of stress management (Table 4).

#### 3.3.9. Life Appreciation

Gender, grade, location of institution, type of institution, both parents' educational level, and residence before being enrolled in school were the variables that were significantly related to spiritual growth in the univariate analyses. Male students had higher levels than female students (*P* = 0.024) on this variable, and higher levels were also observed in freshmen (*P* = 0.000), those students whose institution located in West China (*P* = 0.002), those students whose university is medical university (*P* = 0.000), those who came from urban areas before being enrolled in school (*P* = 0.007), and those whose fathers (*P* = 0.000) and mothers (*P* = 0.000) had completed university education (*P* = 0.000) compared with the other groups. The multiple regression model included three variables (type of institution, grade, and mother's educational level) as predicting dimensions (*R* ^2^ = 0.162; *P* < 0.05), explaining 16.2% of the total variance (Table 4).

## 4. Discussion

The main findings provide insight into two main directions. First, it was determined that the mean scores from all dimensions of healthy lifestyle behaviors, except for the exercise behavior, were at a medium level. Second, healthy lifestyles are modulated by gender, grade, father's level of education, and type of institution when controlling for other social and demographical variables. 

A sedentary lifestyle is a common and serious problem among university students. Compared to young adults in general, the pressure of work is so severe for university students that much of their time and energy is likely to be occupied with their studies. On the other hand, the popularization of computers and the Internet may provide more choices of entertainment and reduce interest in exercise. Lack of exercise facilities is also a major reason why university students do not participate actively in exercise. This result is similar to those obtained in other studies [24, 33, 34]. The previous investigations in Taiwan [35, 36] and Hong Kong [14] found that the mean scores on the health-responsibility and exercise behavior dimensions were lower than the average level; other dimensions were at a medium level. In an educational study carried out with university students, it was established that the mean scores obtained from all domains at baseline, excluding the health responsibility, were also at a medium level [37]. This difference on the score of health-responsibility dimension is considered to arise possibly from the dissimilarity between sociocultural structures and enhancement of health consciousness with time.

We found that female students display an overall healthier profile, whereas it was reported in Hacıhasanoğlu et al.'s and Peltzer's study [24, 38]. It was determined in this study that female students were more likely to take a regular behavior, nutrition behavior and health responsibility, and showed more confidence than male students in the social support dimension. Male students exercise more frequently and manage their stress better than female students but more likely to take a health risk behavior than female students. This result shows similarity with those obtained from some studies conducted in university students [39, 40], although it differs from the results of some studies [34, 41]. It was also reported in some studies [24, 40, 42] that score average of female students was higher than that of male students in the subscales of self-actualization, health responsibility and nutrition, and interpersonal relations, and physical activity score average was higher in male students compared with female students [14, 23, 43]. Unlike the current research, it was determined in the study that 44 female students were more willing to practice healthy life activities compared with male students and their nutrition habits were better, but they were more stressful. In addition, no significant difference was determined between gender and self-actualization, and interpersonal relations and stress management in Hacıhasanoğlu et al.'s study [24]. Ünalan et al. [39] also reported no significant difference between gender and health responsibility, and interpersonal relations and stress management. These results demonstrate that gender is not always determinant in adopting or maintaining better healthy lifestyle behaviors; yet, female students are better in nutrition, health responsibility and interpersonal relations, whereas male students are better in performing physical activities.

Alpar et al. [44] reported a significant difference between the university students' score averages except nutrition over the time period from their first year at university to their graduation year. In our study, it was detected that total HLSUS scores were better among freshmen than other groups, probably because there is no much workload and stress in the freshmen stage. In terms of exercise behavior, regular behavior, health responsibility, social support, stress management, and life appreciation, this study revealed that junior students were far more capable than senior students, which may be because the senior students are engaged in coping with increasing workload and employment stress and had less enthusiasm for university life owing to a longer time of sensitization. There is no difference among grades in nutrition behavior, probably because almost all students have dinner in the canteen. Previous studies on Turkey university students [23, 24] reported that health promoting behaviors score averages of students increased in direct proportion to the increase in their grade levels but not to the analysis of the mutual interference factors of healthy lifestyles by using multivariate analyses.

In all aspects of healthy lifestyle, the university students in the medical university are better than students in the three-year college and comprehensive university, which may be because training of medical curriculums make the medical students pay more attention to adopt healthy lifestyle. It also was reported in the study by Can et al. [45] that the nursing students had more positive health-promoting lifestyles than those of the nonnursing students. The result also suggests the importance of health education for university students which aims to promote healthy lifestyle.

Exercise behavior and nutrition behavior score averages of students and their families were observed to have increased as their level of high income, while regular behaviour and health risk behaviour score averages of students and their families were observed to have decreased as their level of high income. No statistically significant difference was found between family monthly income and the total score average of HLSUS. Can et al. [45] reported those students' total score average of healthy lifestyle behaviours and score averages of subscales of physical activity, nutrition, and interpersonal relations increased with the increase in their level of income. It was also detected in other studies that students' healthy lifestyle behaviour score average increased together with the increase in their family income [34, 40].

In addition, it was found that students' total score averages of healthy lifestyle behaviors and score averages of dimensions of regular behavior, nutrition behavior, health responsibility, and social support increased with the increase in fathers' education level, and score average of stress management and life appreciation increased with the increase in mothers' education level. Ayaz et al. [33] reported that total score average of students' healthy lifestyle behavior scale and score average of health responsibility subscale increased with the increase in mothers' education level. Students' total score average of healthy lifestyle behaviors and score averages of subscales were detected to have increased with the increase in fathers' education level, and this increase was found to be significant in all areas except interpersonal relations. Ulla Díez and Pérez-Fortis[23] found that total score average of students' healthy lifestyle behavior scale and score average of nutrition, physical activity, stress management, and interpersonal relations subscale increased with the increase in mothers' education level. Students' total score average of healthy lifestyle behaviors and score averages of subscales were detected to have increased with the increase in fathers' education level, except nutrition, physical activity, and health responsibility. Tuğut and Bekar [46] also reported a statistically significant difference between fathers' education level and health perception score averages. These different results demonstrate an effect of maternal education over healthy lifestyles of university students, and the difference is considered to arise possibly from the dissimilarity between sociocultural structures.

Nevertheless, some limitations of this study include the following aspects. First, no detailed information about nonresponders was collected. However, the high response rate limited the effect of any bias due to missing information on nonrespondents. Second, although the interviewers received uniform training, their explanations of questionnaire items may have influenced the results. Third, as shown in the tables, even though the regression models are statistically significant, the explained amount of variance is considerably low. Thus, further studies should be conducted in multiple global settings to evaluate university students' healthy lifestyles and associated factors more fully, before the findings are applied widely to the establishment of health-promoting interventions.

## 5. Conclusion

The main findings of this study revealed that a high percentage of university students do not exhibit healthy lifestyles, and these can be predicted to some extent by social characteristics. These results obtained here provide relevant information for future actions. To more effectively reduce chronic illnesses and improve population health, health education programs should be planned to stimulate the interests of different students according to their socio-demographic characteristics.

## Figures and Tables

**Table 1:** Socio-demographic characteristics of the sample (*n* = 4,908).

Socio-demographic characteristics	Frequencies *n*	Percentage %
Gender		
Male	2636	53.71
Female	2272	46.29
Information missing	0	0.00
Grade		
Freshmen	2356	48.00
Sophomores	1285	26.18
Junior students and above	1267	25.81
Information missing	0	0.00
Location of institution		
East China	2843	57.93
Midland	722	14.71
West China	1343	27.36
Information missing	0	0.00
Type of institution		
Three-Year college	1426	29.05
Medical university	1393	28.38
Comprehensive university	2089	42.56
Information missing	0	0.00
Father's educational level		
Primary	684	13.94
Secondary	2996	61.04
University	1209	24.63
Information missing	19	0.39
Mother's educational level		
Primary	1298	26.45
Secondary	2717	55.36
University	858	17.48
Information missing	35	0.71
Residence before enrolled in school		
Rural areas	2187	44.56
Urban areas	1485	30.26
Towns	1229	25.04
Information missing	7	0.14
Family monthly income (RMB)		
0~	1964	40.02
2000~	1952	39.77
5000~	698	14.22
10000~	199	4.05
Information missing	95	1.94

**Table 2:** Distribution and healthy lifestyles scale scores obtained by university students (*n* = 4908).

HLSUS and dimension	Range of obtainable scores (min. and max.)	Range of scores obtained (min. and max.)	$\bar{x}\pm s$
Total HLSUS (38 items)	38–190	65–190	139.84 ± 17.07
Exercise behavior (4 items)	4–20	5–20	11.76 ± 3.00
Regular behavior (4 items)	4–20	4–20	14.72 ± 2.91
Nutrition behavior (4 items)	4–20	4–20	13.87 ± 3.18
Health risk behavior (4 items)	4–20	4–20	14.24 ± 2.37
Health responsibility (6 items)	6–30	6–30	24.81 ± 3.64
Social support (6 items)	6–30	6–30	22.76 ± 4.04
Stress management (5 items)	5–25	5–25	18.39 ± 3.30
Life appreciation (5 items)	5–25	5–25	19.30 ± 3.73

**Table 3:** Comparison values of the socio-demographic characteristics for the healthy lifestyle profile.

Socio-demographic characteristics	Total HLSUS score	Exercise behavior	Regular behavior	Nutrition behavior	Health risk behavior	Health responsibility	Social support	Stress management	Life appreciation
	mean (SD)	mean (SD)	mean (SD)	mean (SD)	mean (SD)	mean (SD)	mean (SD)	mean (SD)	mean (SD)
Gender									
Male	139.70 (19.05)	12.75 (3.31)	14.52 (3.12)	13.92 (3.16)	13.75 (2.68)	24.34 (4.04)	22.31 (4.27)	18.68 (3.44)	19.43 (3.96)
Female	139.97 (15.15)	10.90 (2.39)	14.89 (2.70)	13.83 (3.21)	14.66 (1.98)	25.22 (3.20)	23.15 (3.78)	18.14 (3.19)	19.19 (3.51)
*P* value	0.589	**0.000**	**0.000**	0.303	**0.000**	**0.000**	**0.000**	**0.000**	**0.024**
Grade									
Freshmen	141.29 (17.67)	12.35 (3.29)	14.87 (3.00)	13.72 (3.21)	14.18 (2.61)	25.02 (3.61)	22.92 (4.10)	18.53 (3.39)	19.70 (3.81)
Sophomores	138.00 (16.80)	11.08 (2.53)	14.57 (2.83)	13.79 (3.29)	14.39 (2.11)	24.63 (3.60)	22.55 (4.11)	18.10 (3.40)	18.91 (3.61)
Junior students and above	139.03 (15.93)	11.33 (2.64)	14.58 (2.81)	14.23 (3.01)	14.21 (2.15)	24.61 (3.71)	22.69 (3.83)	18.42 (3.07)	18.95 (3.62)
*P* value	**0.000**	**0.000**	**0.002**	**0.000**	**0.031**	**0.001**	**0.023**	**0.001**	**0.000**
Location of institution									
East China	139.74 ± 17.16	11.44 (2.59)	14.58 (2.88)	14.30 (3.12)	14.10 (2.30)	24.76 (3.80)	22.80 (4.07)	18.60 (3.32)	19.17 (3.76)
Midland	138.27 ± 15.23	10.78 (2.42)	14.70 (2.76)	13.36 (3.22)	14.78 (2.13)	24.84 (3.26)	22.68 (3.87)	17.88 (3.18)	19.25 (3.48)
West China	140.90 ± 17.72	12.96 (3.66)	15.01 (3.02)	13.22 (3.15)	14.25 (2.60)	24.92 (3.48)	22.72 (4.06)	18.21 (3.35)	19.60 (3.77)
*P* value	**0.003**	**0.000**	**0.000**	**0.000**	**0.000**	**0.394**	**0.727**	**0.000**	**0.002**
Type of institution									
Medical university	144.07 (15.64)	12.95 (3.60)	15.38 (2.70)	14.49 (3.02)	14.28 (2.06)	25.17 (3.37)	23.23 (3.65)	18.94 (2.92)	19.62 (3.40)
Three-Year college	136.88 (16.87)	11.11 (2.49)	14.40 (2.94)	13.15 (3.21)	14.34 (2.62)	24.56 (3.61)	22.39 (4.14)	17.69 (3.39)	19.24 (3.76)
Comprehensive university	139.05 (17.57)	11.40 (2.63)	14.49 (2.95)	13.95 (3.18)	14.15 (2.39)	24.74 (3.81)	22.71 (4.17)	18.49 (3.43)	19.12 (3.90)
*P* value	**0.000**	**0.000**	**0.000**	**0.000**	0.057	**0.000**	**0.000**	**0.000**	**0.000**
Father's educational level									
Primary	136.56 (17.66)	11.50 (2.84)	14.44 (2.90)	13.24 (3.10)	14.40 (2.32)	24.07 (3.84)	22.14 (4.25)	17.94 (3.43)	18.82 (3.87)
Secondary	139.75 (16.33)	11.66 (2.94)	14.75 (2.83)	13.82 (3.15)	14.34 (2.31)	24.85 (3.50)	22.71 (3.91)	18.34 (3.21)	19.29 (3.61)
University	141.97 (18.20)	12.14 (3.20)	14.78 (3.09)	14.36 (3.27)	13.91 (2.52)	25.13 (3.80)	23.26 (4.16)	18.77 (3.48)	19.62 (3.89)
*P* value	**0.000**	**0.000**	**0.029**	**0.000**	**0.000**	**0.000**	**0.000**	**0.000**	**0.000**
Mother's educational level									
Primary	137.84 (17.11)	11.48 (2.81)	14.70 (2.84)	13.43 (3.18)	14.53 (2.19)	24.48 (3.66)	22.33 (4.14)	17.99 (3.33)	18.91 (3.79)
Secondary	139.96 (16.42)	11.72 (2.97)	14.70 (2.85)	13.92 (3.13)	14.25 (2.37)	24.89 (3.53)	22.77 (3.90)	18.38 (3.23)	19.33 (3.60)
University	142.50 (18.66)	12.32 (3.30)	14.79 (3.21)	14.39 (3.29)	13.77 (2.58)	25.09 (3.90)	23.39 (4.23)	18.99 (3.49)	19.77 (3.98)
*P* value	**0.000**	**0.000**	0.706	**0.000**	**0.000**	**0.000**	**0.000**	**0.000**	**0.000**
Residence before enrolled in school									
Rural areas	138.97 (16.37)	11.62 (2.84)	14.74 (2.82)	13.56 (3.11)	14.52 (2.32)	24.63 (3.54)	22.48 (3.93)	18.16 (3.24)	19.26 (3.60)
Urban areas	141.78 (17.81)	12.00 (3.16)	14.74 (3.05)	14.33 (3.24)	13.89 (2.42)	25.12 (3.74)	23.30 (4.14)	18.87 (3.37)	19.54 (3.85)
Towns	139.12 (17.14)	11.71 (3.07)	14.65 (2.88)	13.86 (3.18)	14.18 (2.35)	24.78 (3.65)	22.61 (4.02)	18.23 (3.33)	19.10 (3.78)
*P* value	**0.000**	**0.001**	0.674	**0.000**	**0.000**	**0.000**	**0.000**	**0.000**	**0.007**
Family monthly income (RMB)									
0~	139.18 (17.05)	11.66 (2.95)	14.79 (2.91)	13.48 (3.21)	14.51 (2.33)	24.67 (3.64)	22.61 (4.06)	18.13 (3.35)	19.33 (3.72)
2000~	139.95 (17.03)	11.77 (3.06)	14.73 (2.91)	13.94 (3.11)	14.23 (2.27)	24.88 (3.57)	22.79 (3.98)	18.43 (3.25)	19.18 (3.68)
5000~	141.01 (16.82)	11.95 (2.93)	14.58 (2.88)	14.47 (3.18)	13.82 (2.49)	24.92 (3.74)	23.03 (4.00)	18.80 (3.25)	19.44 (3.73)
10000~	142.58 (17.49)	12.42 (3.07)	14.52 (2.83)	14.92 (2.95)	13.25 (2.86)	25.28 (3.63)	23.31 (4.14)	19.04 (3.47)	19.85 (3.99)
*P* value	**0.010**	**0.002**	0.307	**0.000**	**0.000**	0.063	**0.022**	**0.000**	0.059

**Table 4:** Standardized regression coefficients of socio-demographic variables on healthy lifestyles for university students (*n* = 4908).

Socio-demographic characteristics	Total HLSUS score	Exercise behavior	Regular behavior	Nutrition behavior	Health risk behavior	Health responsibility	Social support	Stress management	Life appreciation
Gender	−0.151	0.221	−0.108		−0.182	−0.147	−0.136	0.051	
Grade									
Junior students and above									
Freshmen	−0.107	−0.089	−0.076			−0.204	−0.059	−0.178	−0.095
Sophomores		−0.054					−0.122		
Type of institution									
Medical university									
Three-Year college	−0.187	−0.055	−0.087	−0.292		−0.033		−0.288	−0.092
Comprehensive university	−0.194		−0.100	−0.168				−0.227	−0.132
Family monthly income (RMB)									
10000~									
0~		0.105		0.076	−0.106				
2000~			−0.182						
5000~					−0.011				
Father's educational level									
University									
Primary	0.126		0.143	0.058		0.064	0.131		
Secondary	0.188		0.156	0.069		0.089	0.201		
Mother's educational level									
University									
Primary					−0.034			0.078	0.063
Secondary					−0.106			0.145	0.123
*R* ^2^	0.3202	0.4173	0.2104	0.2247	0.1936	0.0922	0.2013	0.2181	0.1620

Note: The data in Table 3 is the standardized regression coefficients (*β*), and value of *P* is less than 0.05.

Variables: Type of institution (three-year college = 1, others = 0; comprehensive university = 1, others = 0); gender (female = 1, male = 0); learn health education curriculum status (have studied = 1, have not studied = 0); grade (freshmen = 1, others = 0; sophomores = 1, others = 0); father's educational level (primary = 1, others = 0; secondary = 1, others = 0); mother's educational level (primary = 1, others = 0; secondary = 1, others = 0); family monthly income (RMB) (0~ = 1, others = 0; 2000~ = 1, others = 0; 5000~ = 1, others = 0).
